# Early response to JAK inhibitors on central sensitization and pain catastrophizing in patients with active rheumatoid arthritis

**DOI:** 10.1007/s10787-022-00995-z

**Published:** 2022-05-03

**Authors:** Fausto Salaffi, Marina Carotti, Sonia Farah, Luca Ceccarelli, Andrea Giovagnoni, Marco Di Carlo

**Affiliations:** 1 Rheumatology Clinic, Università Politecnica Delle Marche, Ospedale “C. Urbani”, Jesi, Ancona Italy; 2 Department of Radiological Sciences, Radiology Clinic, Azienda Ospedaliero-Universitaria, Ospedali Riuniti di Ancona, Ancona, AN Italy; 3 Department of Interventional and Diagnostic Radiology, Azienda Ospedaliero-Universitaria Sant’Anna, Ferrara, Italy

**Keywords:** Rheumatoid arthritis, Central sensitization, Pain catastrophizing, JAK inhibitors

## Abstract

**Objectives:**

To evaluate the effect of 4 weeks of treatment with Janus kinase inhibitors (JAKis) on central sensitization (CS) and pain catastrophizing, and to determine the pain-related variables predictive of disease activity improvement, in patients with active rheumatoid arthritis (RA).

**Methods:**

Consecutive RA patients with active disease starting a JAKi have been enrolled in this prospective observational study. Patients have been assessed at baseline and after 4 weeks of treatment. The evaluation was comprehensive of disease activity [Simplified Disease Activity Index (SDAI) and ultrasonographic (US) score] and of questionnaires aimed at investigating primarily CS [Central Sensitization Inventory (CSI)] and pain catastrophizing [Pain Catastrophizing Scale (PCS)]. Differences (*Δ* values) between the final and baseline were studied with the *t* test, *Δ* values of the variables were correlated with each other using Pearson’s test, and predictor variables for improvement in SDAI were also investigated using multivariate regression analysis.

**Results:**

A total of 115 patients were evaluated. Overall, all variables demonstrated significant improvement between baseline and final except the US score. In particular, CSI decreased from 36.73 to 32.57 (*p* < 0.0001), PCS decreased from 32.46 to 28.72 (*p* = 0.0001). *Δ*SDAI showed a significant correlation with both *Δ*PCS and *Δ*CSI (*r* = 0.466 and 0.386, respectively, *p* < 0.0001). *Δ*PCS was the only variable predictive of an improvement in SDAI (coefficient = 0.500, *p* = 0.0224).

**Conclusion:**

JAKis would appear to have a positive effect on pain-related variables, particularly CS and pain catastrophizing, for the genesis of which extra-synovial mechanisms are responsible.

## Introduction

Persistent pain is a common problem for patients with rheumatoid arthritis (RA), despite a good control of synovitis (Lee et al. 2011; Malaviya and Ostör 2012; Kulkarni and Singh 2008; Pollard et al. 2006). With the earlier start of disease-modifying therapies and the development of targeted immunomodulating agents, more and more patients with RA can reach low disease activity or remission (Lee et al. 2009; Keystone et al. 2010). Although most patients report that inflammatory joint disease is under control, pain perception can be moderate to severe. In patients with RA, it is, therefore, common to deal with subjects who show a clear discrepancy between objective signs of inflammation and pain (and disability), and erroneously one might consider a condition in remission as undergoing high disease activity (Welsing et al. 2005; Taylor et al. 2010).

Underlying the persistence and increased pain symptoms beyond inflammatory genesis in patients with RA are recognized mechanisms of central sensitization (CS), which are documented in approximately 20–30% of patients. CS is an abnormal spinal and supraspinal pain processing that increases nociceptive afferent input. Neuroplastic changes in sensory pathways at both the peripheral and central levels are linked to pain sensitization.

It manifests in the body as a hypersensitivity to pain that extends beyond the area from which the pain itself originates (Woolf 2011; Gebhart 2004). CS, on the other hand, may make pain worse in people with active RA and predicts poor treatment outcomes in a wide range of patient groups (Hammer et al. 2018). CS has been able to explain many of the changes in pain sensitivity that happen in acute and chronic clinical pain settings (Edwards et al. 2011).

Catastrophizing, which is a strong predictor of pain, has also been linked to CS, but few studies have investigated possible interactions between catastrophizing and CS (Hammer et al. 2018). Pain catastrophizing is the exaggerated tendency to repeatedly think about one's pain and imagine the worst that could happen. In chronic musculoskeletal diseases, pain catastrophizing has a major impact on pain experience and pain-related outcomes, such as disability and hospital length of stays (Edwards et al. 2011; Gauthier et al. 2006; Riddle et al. 2010). Whether pain catastrophizing is a psychological trait that is modifiable depending on the patient's clinical situation or is a persistent feature there is no uniformity of view (Quartana et al. 2009). Recently, a study of patients who underwent spinal surgery for lumbar spinal stenosis found that pain catastrophizing largely decreased 3 years after surgery (Kim et al. 2018). Similarly, a long-term study of patients with chronic anterior knee pain revealed that at 6 months after physical or surgical treatment, pain catastrophizing was reduced (Doménech et al. 2014). These observations show that resolving the musculoskeletal problems responsible for pain can also have positive repercussions on certain psychological traits. Specifically, pain catastrophizing would appear to improve with relief of the cause of the pain itself. However, few studies demonstrate how treating the underlying condition responsible for the pain also improves pain catastrophizing.

The Janus kinase/signal transducers and activators of transcription (JAK/STAT) signaling pathway has been linked to the signaling of several cytokines, such as interleukin (IL)-6, IL-12, IL-23, and interferons (IFNs) that play a role in the pathophysiology of RA. In randomized clinical trials, JAK inhibitors (JAKis) have been found to be very effective at reducing pain and other patient-reported outcomes (Rocha et al. 2021; George et al. 2020). The JAK/STAT pathway would appear to be important in the pathophysiology of CS-related neuroplasticity mechanisms. This pathway is also present at the level of the dorsal root ganglia and spinal cord, may play a role in the sensitization of nociceptors caused by joint inflammation (Vieira et al. 2016).

Starting from these assumptions, the objectives of this study were to evaluate the effect of 4 weeks of treatment with JAKis on CS, pain catastrophizing, and to determine the pain-related variables predictive of disease activity improvement, in patients with active RA.

## Methods

### Patients, inclusion and exclusion criteria

From April 2021 to February 2022, active RA patients eligible for treatment with JAKis, were prospectively enrolled in this prospective observational study at the Rheumatology Clinic of the Università Politecnica delle Marche, “Carlo Urbani” Hospital, Jesi (Ancona), Italy. To be included in the study, patients had to meet the 2010 American College of Rheumatology (ACR)—European League Against Rheumatism (EULAR) classification criteria for RA and be 18 or older (Aletaha et al. 2010). Active disease was defined by > 6 painful joints (68-joint count) and > 6 swollen joints (66-joint count) and by a C-reactive protein level of > 0.7 mg/dl. Patients were also required to have evidence of > 3 distinct joint erosions on hand, wrist and foot radiographs as determined by the radiologists (MC and LC), or, if radiographic evidence of joint erosions was unavailable, the presence of rheumatoid factor (RF) or anti-citrullinated protein/peptide antibody (ACPA) positivity. Stable doses of methotrexate (MTX) were required (15–25 mg weekly for > 6 weeks; stable doses > 15 mg was allowed only if there were safety issues at higher doses), JAKi monotherapy was permitted in patients intolerant to MTX. Stable doses of low-dose corticosteroids (< 10 mg/day prednisone or equivalent) and nonsteroidal anti-inflammatory drugs (NSAIDs) were allowed. Prior use of biologic or non-biologic DMARDs was permitted.

Exclusion criteria were: hemoglobin < 9.0 g/dl, hematocrit < 30%, white blood cell count < 3.0 × 10^9^/l, absolute neutrophil count < 1.2 × 10^9^/l, or platelet count < 100 × 10^9^/l, estimated glomerular filtration rate < 40 ml/min (Cockcroft-Gault calculation), aspartate aminotransferase (AST) or alanine aminotransferase (ALT) levels > 1.5 the upper limit of normal (ULN); recent, current, or chronic infections (including inadequately treated hepatitis B or C or Mycobacterium tuberculosis infection); or active neoplasms or lymphoproliferative disorders; coexisting diseases of the central or peripheral nervous systems (Alzheimer’s disease or other dementias, Parkinson’s disease, motor neuron disease, polyneuropathy, multiple sclerosis, spinal lesions); coexisting fibromyalgia (diagnosed according to the 2016 ACR criteria) or concomitant treatment with centrally acting pain medications (e.g., opioids, nortriptyline, duloxetine, gabapentin, and pregabalin).

All patients received a drug targeting the JAK/STAT signaling pathway, including tofacitinib, baricitinib, upadacitinib, and filgotinib. Tofacitinib is a pan JAKi with greater selectivity for JAK1/JAK3 and minor activity on JAK2 and TYK2. Baricitinib is a JAK1/JAK2 inhibitor with moderate activity against TYK2 and minimal activity against JAK3 (Clark et al. 2014; Genovese et al. 2016). Upadacitinib and filgotinib aims to solely target the JAK1 pathway (Yamaoka 2016)*.*

### Composite disease activity assessment and ultrasonographic examination

Because no one measure can serve as a “gold standard” quantifying disease activity in RA requires a pooled index comprising several different parameters. In this study, the Simplified Disease Activity Index (SDAI) and the ultrasonography (US) score were employed. The SDAI is the algebraic sum of 5 untransformed, unweighted variables, including 28 swollen joint count (SJC) and 28 tender joint count (TJC), patient global assessment (PGA) and evaluator global assessment (EGA) of disease activity on 0–10 numerical rating scales (NRS), and C-reactive protein (CRP) in mg/dl. SDAI range is from 0 to 86, and remission, low, and moderate disease activity have predefined upper limits of 3.3, 11 and 26, respectively (Smolen et al. 2003).

The US scoring system has already been employed to calculate composite indices (Salaffi et al. 2018a, 2010), and evaluated the radiocarpal, 2nd and 3rd metacarpophalangeal, and 2nd and 3rd proximal interphalangeal joints. Dorsal scans were performed to identify the presence of signs of synovitis in both grey scale (GS) and power Doppler (PD) signal (Ohrndorf and Backhaus 2013). According to OMERACT definitions, synovitis was characterized as a grade of at least 1 on both GS and PD (Wakefield et al. 2005). The results of the US assessment has been weighted according to Thompson's articular index (Thompson et al. 1987), and then normalized on a 0–10 scale (Salaffi et al. 2018a). The US assessment was performed with a MyLab Class C (Esaote S.p.A., Genoa, Italy) equipped with a 6–18 MHz linear probe.

### Questionnaires

Patients were studied with a patient-reported questionnaire package aimed at investigating the study objectives. The Central Sensitization Inventory (CSI) was used as an estimate of central sensitization of symptoms (Mayer et al. 2012) and the Pain Catastrophizing Scale (PCS) for assessing catastrophizing cognitions (Sullivan et al. 1995). The assessment of pain features was also completed with the painDETECT Questionnaire (PDQ) for evaluating the neuropathic pain features (Freynhagen et al. 2006) and the Semantic Questionnaire for Rheumatology (SQR) to estimate pain sensitivity (Nolli et al. 1988; Cimmino et al. 2004). A brief description of the questionnaires is provided below.

#### Central Sensitization Inventory (CSI)

The CSI is a screening tool for detecting signs of CS syndrome (CSS) and is composed of two parts. Part A has 25 questions concerning CS symptoms, graded on a 5-point Likert scale from 0 to 4. Higher overall scores indicate more CS symptoms. A cut-off point of 40 out of 100 indicates the presence of CSS (Mayer et al. 2012; Neblett et al. 2013, 2015). The CSS severity categories are subclinical (0–29), mild (30–39), moderate (40–49), severe (50–59), and extreme (60–100) (Neblett et al. 2013). Part B assesses previously identified CS-related diseases, which were not examined in this research. The Italian CSI showed excellent construct validity, a good discriminative power and excellent test–retest reliability (Chiarotto et al. 2018).

#### Pain Catastrophizing Scale (PCS)

The PCS is a 13-item tool that evaluates catastrophic thinking connected to pain assessing rumination, amplification, and helplessness. The PCS total score is obtained by adding all 13 replies on 5-point scales with 0 being “never” and 4 being “always”. A total PCS score of 30 reflects a clinically meaningful degree of catastrophizing (Sullivan et al. 1995). It has been integrated into the evaluation routine of pain clinics and rehabilitation centers throughout North America and Europe, as well as the chronic pain registry (Granan et al. 2019). PCS Italian version validated, psychometric properties comparable to other versions (Monticone et al. 2012).

#### PainDETECT Questionnaire (PDQ)

PDQ contains nine items, of which seven related to sensory responses and two to the temporal and spatial characteristics of the pain pattern. The algebraic sum of the seven items category scores (from 0 for never to 5 for very strongly) and the scores for temporal (− 1 to + 1) and spatial qualities (0 or + 2) yield to a score ranging from − 1 to 38. A score < 12 indicates that the pain is unlikely to be neuropathic, a score > 19 indicates that pain is likely to be neuropathic, and a score between 13 and 18 indicates a slight chance of neuropathic component (Freynhagen et al. 2006). The Italian PDQ demonstrated high test–retest reliability and discrimination between nociceptive and neuropathic pain (Migliore et al. 2021).

#### Semantic Questionnaire for Rheumatology (SQR)

The SQR is made of 23 terms derived from the McGill Pain Questionnaire (MPQ) (Melzack and Katz 2001), 11 descriptors come from the sensory component, 10 from the emotional component, and 2 from the evaluative section. The final score is between 0 and 51. The patient is instructed to go through this list of pain descriptors and circle those that best match his or her present pain condition. The ranks for the descriptors are added for each section or dimension of the SQR, and a cumulative total score [Pain Rating Index (PRI)] can be computed (Nolli et al. 1988; Cimmino et al. 2004). For the purposes of this study, the PRI-SQR has been used.

### Statistical analysis

All the data were entered into a Microsoft Excel database and analyzed using MedCalc^®^ 64-bit version 19.0.1.0. (MedCalc Software, Mariakerke, Belgium). The Kolmogorov–Smirnov test was used to determine if the distribution was normal. A paired *t* test was performed to study the scores changes between baseline and after 4 weeks of JAKis administration. The degree of correlation between the difference (*Δ*, final minus baseline values) of the variables (CSI, PCS, PDQ, PRI-SQR, SDAI and US score) was measured using Pearson’s *r*. The correlation strength was classified as very mild (0.00–0.19), weak (0.20–0.39), moderate (0.40–0.59), strong (0.60–0.79), and very strong (0.80–1.00) for r values. To evaluate the difference in clinical outcomes among the various JAKis, a one-way analysis of variance (ANOVA) was performed distinguishing patients into seven categories, respectively, single molecule monotherapy (tofacitinib, baricitinib and upadacitinib, for filgotinib no patients in monotherapy were included) and their association with MTX (tofacitinib + MTX, baricitinib + MTX, upadacitinib + MTX and figotinib + MTX). Finally, to determine predictors of improved disease activity, a multivariate analysis was conducted considering the reduction in SDAI as the dependent variable and the changes of pain-related indices (*Δ*CSI, *Δ*PCS, *Δ*PDQ, *Δ*PRI-SQR), the changes of sonographic findings (*Δ*US score), laboratory (ACPA titer), and demographic features (age, BMI, duration of disease, level of education) as independent variables.

## Results

### Patient characteristics

The study was completed by 115 patients (84.3% of women), with a mean age of 58.4 years and a mean disease duration of 10.3 years. Before starting a JAKi, the 61.7% of patients was taking a non-biologic DMARD, and the 38.3% was taking a biologic DMARD. Twenty-two patients were treated with tofacitinib monotherapy (5 mg BD), 19 patients received tofacitinib and methotrexate (MTX), 19 patients received baricitinib monotherapy (4 mg OD), 14 patients received baricitinib and MTX, 17 patients received upadacitinib monotherapy (15 mg OD), 16 patients received upadacitinib and MTX, and 8 patients received filgotinib and MTX (200 mg OD).

At baseline, the mean (SD) SDAI was 44.3 (17.4) and CRP levels were 1.5 mg/dl. Overall, the 36.5% showed symptoms of CS (CSI ≥ 40), the 62.6% exceeded the threshold for the presence of pain catastrophizing (PCS > 30), and the 43.5% showed symptoms of neuropathic pain (PDQ ≥ 19) (Table 1).

**Table 1:** Baseline demographic and clinical characteristics

Variable	Mean	SD
Age (years)	58.4	11.6
Gender (% female)	84.3%	
BMI (kg/m^2^)	26.1	4.4
Disease duration (years)	10.3	7.8
Educational level (years)	10.0	3.5
ACPA (titer, U/ml)	274.0	475.8
RF (titer, U/ml)	139.5	207.2
CRP (mg/dl)	1.5	1.6
SJC	6.4	4.1
TJC	10.1	5.2
EGA	6.5	1.7
PGA	7.2	1.8
SDAI	44.3	17.4
US score	5.3	1.4
PDQ	17.7	5.6
PRI-SQR	17.2	6.1
PCS	32.5	9.7
CSI	36.7	12.6

*BMI* body mass index, *ACPA* anti-citrullinated protein/peptide antibody, *RF* rheumatoid factor, *CRP* C-reactive protein, *SJC* swollen joint count, *TJC* tender joint count, *EGA* Evaluator Global Assessment of disease activity, *PGA* = Patient Global Assessment of disease activity, *SDAI* Simplified Disease Activity Index, *US* ultrasound, *PDQ* PainDETECT Questionnaire, *PRI-SQR* Pain Rating Index of the Semantic Questionnaire for Rheumatology, *PCS* Pain Catastrophizing Scale, *CSI* Central Sensitization Inventory

### Changes in disease activity and pain-related variables

After 4 weeks of treatment, 12 patients (10.5%) achieved SDAI remission, and 18 (15.6%) met the criteria for low disease activity (LDA). SDAI decreased from 44.30 to 27.83 (*p* < 0.0001). The US score did not change significantly (5.25 to 5.03; *p* = 0.226).

CSI decreased from 36.73 to 32.57 (*p* < 0.0001), PCS decreased from 32.46 to 28.72 (*p* = 0.0001), PDQ decreased from 17.65 to 15.66 (*p* = 0.0005), and the PRI-SQR decreased from 17.21 to 12.52 (*p* < 0.0001).

### Correlation analysis of differences

**Fig. 1:**
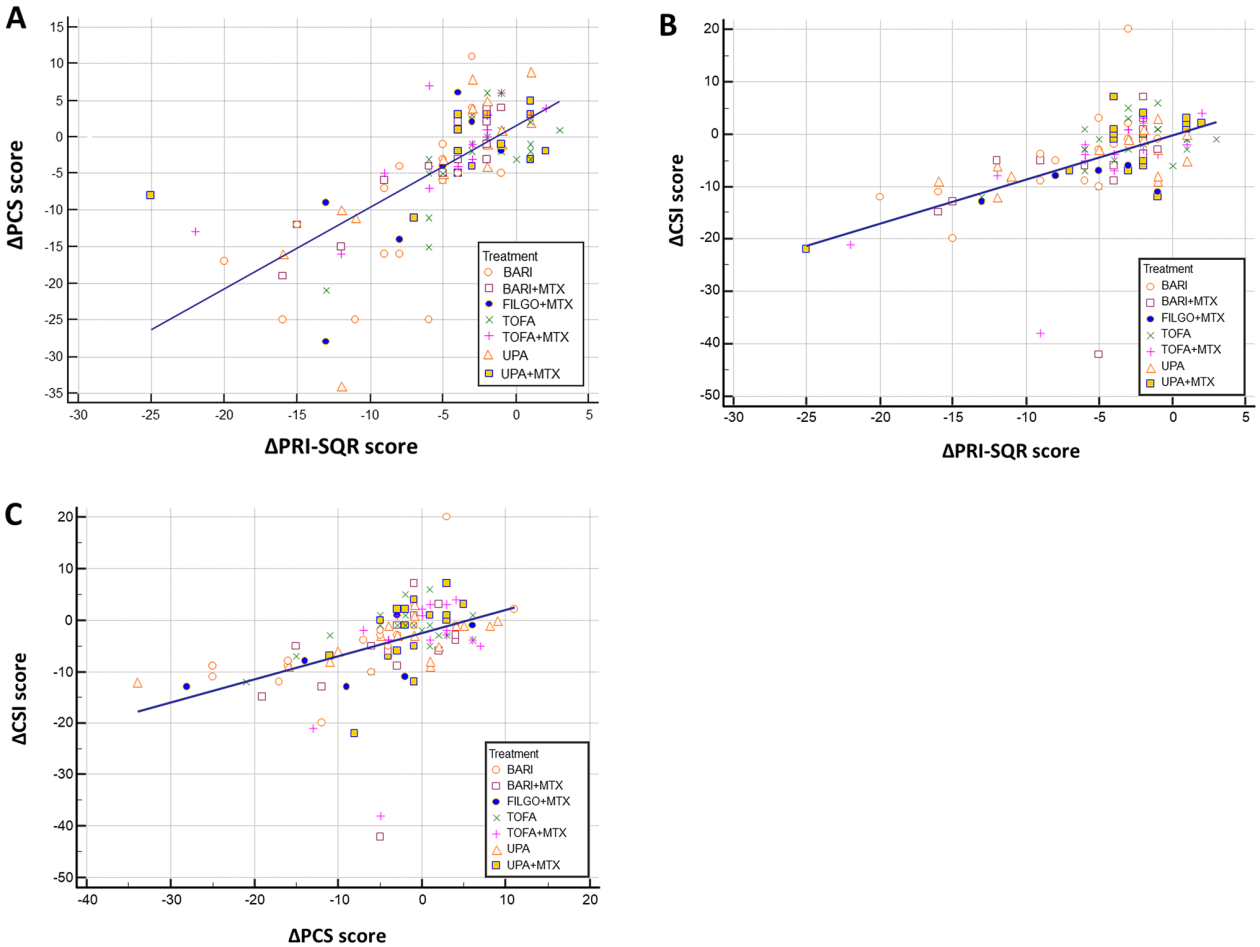
Scatterplot with linear regression lines displaying the relationship between the differences (*Δ* values) in PCS vs PRI-SQR (**a**), CSI vs PRI-SQR (**b**), and CSI vs PCS score (**c**), according to the different treatments. *PCS* Pain Catastrophizing Scale, PRI-*SQR* Pain Rating Index of the Semantic Questionnaire for Rheumatology, *CSI* Central Sensitization Inventory, *BARI* baricitinib, *FILGO* filgotinib, *TOFA* tofacitinib, *UPA* upadacitinib, *MTX* methotrexate

Significant relationships have been found between the *Δ*PCS and the *Δ*PRI-SQR (*r* = 0.714, *p* < 0.0001), between *Δ*CSI and *Δ*PRI-SQR (*r* = 0.571, *p* < 0.0001), and between *Δ*CSI and *Δ*PCS (*r* = 0.475, *p* < 0.0001) (Table 2, Fig. 1). Among other variables, *Δ* SDAI demonstrated to be substantially linked with *Δ*PCS and *Δ*CSI (*r* = 0.466 and 0.386, respectively, *p* < 0.0001), and to a lesser extent with *Δ*US score (*r* = 0.318, *p* = 0.0006) (Table 2).

**Table 2:** Pearson’s correlation coefficient r for differences (*Δ* values) in continuous composite indices of disease activity (SDAI and US score) and patient-reported outcomes (PROs) measures

	*Δ*PCS	*Δ*PDQ	*Δ*PRI-SQR	*Δ*SDAI	*Δ*US score
*Δ*CSI					
*r* *p*	0.475 < 0.0001	0.339 0.0002	0.571 < 0.0001	0.386 < 0.0001	0.246 0.0081
*Δ*PCS					
*r* *p*		0.337 0.0002	0.714 < 0.0001	0.466 < 0.0001	0.336 0.0002
*Δ*PDQ					
*r* *p*			0.344 0.0002	0.265 0.0044	0.220 0.0181
*Δ*PRI-SQR					
*r* *p*				0.427 < 0.0001	0.401 < 0.0001
*Δ*SDAI					
*r* *p*					0.318 0.0006

*CSI* Central Sensitization Inventory, *PCS* Pain Catastrophizing Scale, *PDQ* PainDETECT Questionnaire, *PRI-SQR* Pain Rating Index of the Semantic Questionnaire for Rheumatology, *SDAI* Simplified Disease Activity Index, *US* ultrasound

### Differences among treatments

Considering the distinction of patients into the different treatment categories, the ANOVA did not document statistically significant differences among the various JAKis, whether in monotherapy or in combination with MTX, for either *Δ*CSI (*p* = 0.465), *Δ*PCS (*p* = 0.165), or *Δ*PRI-SQR (*p* = 0.144).

### Multivariate regression analysis

The multivariate regression analysis, conducted using *Δ*SDAI as the dependent variable, revealed *Δ*PCS (coefficient = 0.5009, *p* = 0.0224) as the only associated independent variable (Table 3).

**Table 3:** Multivariate regression analysis, using SDAI (Simplified Disease Activity Index) changes as dependent variable

Independent variables	Coefficient	Std. error	*T*	*p*
(Constant)	− 0.7239			
ACPA (titer)	− 0.0002410	0.002528	− 0.0953	0.9242
Age (years)	− 0.09063	0.1028	− 0.881	0.3803
BMI	− 0.1600	0.2787	− 0.574	0.5671
Disease duration (years)	− 0.1003	0.1611	− 0.623	0.5347
Educational level (years)	− 0.1072	0.3703	− 0.289	0.7728
*Δ*CSI	0.3194	0.1984	1.610	0.1105
*Δ*PRI-SQR	0.07018	0.3726	0.188	0.8509
*Δ*US score	1.2248	0.7024	1.744	0.0842
*Δ*PCS	0.5009	0.2160	2.318	0.0224
*Δ*PDQ	0.1860	0.2271	0.819	0.4146

*ACPA* anti-citrullinated protein/peptide antibody, *BMI* body mass index, *CSI* Central Sensitization Inventory, PRI-*SQR* Pain Rating Index of the Semantic Questionnaire for Rheumatology, *PCS* Pain Catastrophizing Scale, *US* ultrasound, *PDQ* PainDETECT Questionnaire

## Discussion

In this study, it was demonstrated how, in patients with RA, some pain-related centralization and psychological traits are closely related to clinical improvement, intended as disease activity, and that JAKis, because of their distinctive pharmaco-dynamic properties, may have an effect on these extra-synovial aspects of pain. To the best of our knowledge, no study has looked at the relationship between changes in disease activity and changes in CS, pain catastrophizing, and neuropathic pain features that occur with the introduction of a novel treatment targeting the JAK/STAT signaling pathway in active RA patients.

RA has long been thought to be a peripheral inflammatory joint disease, with immunological processes triggering cytokine activation at the synovial level, resulting in joint inflammation, structural joint destruction, and pain. Persistent afferent pain input can lead to synaptic changes and dysregulation in the neurons involved in the pain circuitry of the central nervous system, including the brain and the spinal cord (Zhang and Lee 2018). CS can result from multiple mechanisms, including increased excitatory neurotransmission, diminished inhibitory neurotransmission, or both (Latremoliere and Woolf 2009). Pain hypersensitivity was observed in RA patients using a pain model based on the recording of cortical chemo-somatosensory event-related potentials following painful stimulation of the nasal mucosa (Wendler et al. 2001). RA patients with more than 5 years of symptoms had widespread allodynia to pressure, increased sensitivity to mild touch, and hyperalgesia to benign cold. These patients also demonstrated lower pressure pain thresholds at joint and non-joint locations than healthy controls, as well as pressure allodynia at the thigh (Leffler et al. 2002). Moreover, RA patients showed widespread hyperalgesia to mechanical and thermal stimulation across numerous body regions as compared to controls (Edwards et al. 2009). Patients with RA have been shown to have hyperalgesia, or increased sensitivity to painful stimuli, which is a distinguishing hallmark of fibromyalgia (Leffler et al. 2002; McDermid et al. 1996; Gerecz-Simon et al. 1989). In chronic clinical pain situations, CS offers a mechanistic explanation for many of the temporal, spatial, and threshold variations in pain sensibility, emphasizing the essential role of changes in the central nervous system (CNS) in the determinism of aberrant pain sensitivity. Some of these hyperalgesic reactions may be related to pain catastrophizing. Pain catastrophizing and pain intensity have been correlated in many investigations of RA patients, both cross-sectionally and longitudinally (Holtzman and DeLongis 2007; Covic et al. 2003; Lefebvre and Keefe 2002). Higher pain catastrophizing has been linked to increased CNS sensitization during long-term pain, which might explain the persistent positive connection between pain catastrophizing and pain sensitivity (Edwards et al. 2004). It is also worth mentioning that other elements, such as neuropathic pain features, interact with central sensitization to influence a favorable or negative outcomes (Salaffi et al. 2019).

Catastrophizers have more difficulty in controlling or suppressing their thoughts about pain than do non-catastrophizers. They ruminate more about their pain, and their cognitive and physical abilities are more impacted by the anticipation of pain (Crombez et al. 1998; Van Damme et al. 2002, 2004). There is debate whether pain catastrophizing is a state (temporary and situational) or a trait (constant and enduring). Because pain catastrophizing is a strong predictor of treatment success, it is a good target for multidisciplinary pain-management interventions. It was found that pain catastrophizing was a stable trait that did not change over time in 223 RA patients who were on the same treatment (Keefe et al. 1989). Catastrophizing, on the other hand, was found to have both state and trait components in a 30-day diary study of people with RA (Sturgeon and Zautra 2013). The lack of a link between changes in US score and changes in pain catastrophizing in people with RA raises questions about the relationship between inflammation and catastrophizing in RA. Studies in healthy people have shown that individuals who think of pain as a major problem are more likely to have high levels of IL-6 responsiveness (Edwards et al. 2008). Only one other study looked at the relationship between catastrophizing and measures of inflammation in people with RA (Hammer et al. 2018). Weak relationships were found between the rate of erythrocyte sedimentation and pain catastrophizing, but there were no relationships between the number of swollen joints and pain catastrophizing. People who have changes in their peripheral active synovitis (as measured by US) and changes in pain catastrophizing may have non-inflammatory mechanisms involved, like CS.

Prevention or treatment of CS could be a new and important way to stop or alleviate pain in RA (Roodenrijs et al. 2021). In a primary care setting, symptoms of CS, as measured by the CSI, seemed to be a good predictor of pain-related disability in people with RA, with worse symptoms of CS predicting more pain-related disability (Tanaka et al. 2019). In patients with knee osteoarthritis, preoperative CS lasted 2 years after total knee arthroplasty, and patients with preoperative CS had a worse quality of life, worse disability, and a greater dissatisfaction with their treatment at 2 years after surgery (Koh et al. 2020). Another study found that 664 patients who underwent spinal fusion surgery had worse quality of life and stayed in the hospital longer if they had symptoms of CS before the surgery, and for every 10-point rise in CSI, the length of hospital stays increased by 6.4% (Bennett et al. 2017). One of the main theories about how the CS develops says that this is caused by an inflammatory reaction in the CNS. This includes the activation of astrocytes and microglial cells, the production of cytokines, inflammatory mediators, and neurotrophic factors. As chronic pain and CS progress, the intracellular JAK/STAT pathway plays a major role (Salaffi et al. 2018b).

The results of this study showed no significant differences in the clinical outcomes studied between individual JAK is, and the effect on CS would therefore appear to be attributable to a class effect. However, given the limited number of patients in certain treatments, this aspect needs further investigation. Inhibition of the JAK/STAT pathway is particularly complex and results in a number of pleiotropic effects. The JAK family has more than 90 tyrosine kinases, of which four play an important role in the mechanism of signal transduction in inflammatory diseases, namely JAK1, JAK2, JAK3, and TYK2. The JAK is currently licensed for use in RA are tofacitinib (JAK1/JAK3 inhibitor), baricitinib (JAK1/JAK2 inhibitor), upadacitinib (JAK1 inhibitor), and filgotinib (JAK1 inhibitor). Most cytokines exert their function by activating the JAK/STAT pathway after binding type I/II cytokine receptor. The three main cytokines that do not activate this mechanism are tumor necrosis factor (TNF), IL-1 and IL-17 (O'Shea et al. 2013).

The main cytokine involved in the CS of pain during RA seems to be IL-6, capable of activating both JAK1 and JAK2. Its actions take place both at synovial and dorsal root ganglia level (Choy and Calabrese 2018). Granulocyte macrophage colony-stimulating factor (GM-CSF), which predominantly activates JAK2, is a cytokine that has been documented to play a role in mediating arthritic pain in addition to myeloid line cells (Cook et al. 2013). IL-3, belonging to the hematopoietic growth factor line (also known as multi-CSF), also exerts a pro-nociceptive role in inflammatory pain by predominantly activating JAK2. IL-4, which acts through JAK1–JAK3, is able to activate sensory neurons (Cook et al. 2018).

The JAK/STAT pathway could also modulate anti-nociceptive mechanisms since IL-10, a potent anti-inflammatory cytokine, carries out its action through the JAK/STAT pathway, activating JAK1, JAK2 and TYK2 (Baral et al. 2019).

This study has some limitations. First, the decline in disease activity over the course of the study was considered to be due to the medications administered during the time period considered. However, because this was a prospective observational study rather than a randomized controlled trial, we are unable to rule out the Hawthorne effect, which is defined as the occurrence of symptom improvement as a result of having been noted (Berthelot et al. 2019). Second, the small sample size may have caused some instability in the results, and a larger sample size would be needed for confounders’ correction to identify predictors for SDAI outcome and to examine changes in CS and pain catastrophizing in response to JAKis treatment.

In conclusion, there is substantial evidence that CS and pain catastrophizing are prevalent in many active RA patients. Because of the influence of CS and pain catastrophizing, centrally acting immunomodulatory drugs such as JAKis could result in a rapid analgesic effect. Next to pharmacological therapy, effective non-pharmacological approaches on CS and pain catastrophizing would also be desirable to be more integrated in the therapy of patients with RA (Nijs et al. 2019). Further studies of pain phenotyping are needed to identify personalized targets that can be used to guide therapy.

## Data Availability

The datasets generated during and/or analysed during the current study are available from the corresponding author on reasonable request.
